# Supplementary material for: Mycetoma as a major cause of limb amputation in Northeastern Ethiopia: A facility-based retrospective study
Source: PLoS Negl Trop Dis. 2026 May 6;20(5):e0013886. doi: 10.1371/journal.pntd.0013886 (PMC13197063; doi:10.1371/journal.pntd.0013886)
Supplement: Attachment — Submitted filename: ethiopia mycetoma comment.docx [file pntd.0013886.s002.docx]

Method:

To get more reliable data, suggestion to expand the study duration for 5 years

How other differential diagnosis of mycetoma e.g., malignancy and infections were ruled out?

What were the process for sending diagnostic tests? Does histopathology and culture of the amputee specimens were done to confirm the diagnosis?

Mention the duration of medical options as a variable in method?

Explain the severity impairment criteria in methods, how it was classified as sever, Moderate and mild.

Specify in the methods what were the clinical factors for limb loss? e.g., Bone involvement, massive and advance lesion, failed medical treatment, severe superimposed bacterial infections, patient disability, recurrence after surgery.
