# Supplementary material for: Mycetoma as a major cause of limb amputation in Northeastern Ethiopia: A facility-based retrospective study
Source: PLoS Negl Trop Dis. 2026 May 6;20(5):e0013886. doi: 10.1371/journal.pntd.0013886 (PMC13197063; doi:10.1371/journal.pntd.0013886)
Supplement: Attachment — Submitted filename: POINT BY POINT RESPONSE TO REVIEWERS2522026.docx [file pntd.0013886.s003.docx]

**POINT BY POINT RESPONSE TO REVIEWERS**

We sincerely thank the reviewer for the constructive and insightful comments. We have carefully revised the manuscript to improve clarity, methodological transparency, and completeness. Our point-by-point responses are provided below.

**METHOD:**

**Comment 1:** “To get more reliable data, suggestion to expand the study duration for 5 years.”

**Response:** We appreciate this suggestion. The original objective was to collect five years of retrospective data. However, due to limitations in the hospital’s paper-based filing system, complete patient records for the earlier years were not retrievable. Specifically, files from the intended earlier years were missing or incomplete. Therefore, the study period was restricted to September 2022–August 2025, during which registry documentation was consistently available.

To ensure transparency, we have clarified this in the Methods section and acknowledged it as a limitation in the Discussion.

Manuscript revision made to clarify in Methods under Study Design and added explanation in Limitations subsection of Discussion.

**Comment 2:** “How other differential diagnosis of mycetoma e.g., malignancy and infections were ruled out?”

**Response:** In routine clinical practice at Dubti Referral Hospital, patients were evaluated by physicians based on clinical presentation. When indicated, imaging (primarily X-ray) and occasionally biopsy were performed to rule out differential diagnoses such as malignancy, chronic osteomyelitis, or other soft tissue infections. However, given the retrospective design and limitations of documentation, we cannot systematically describe the decision-making algorithm for each case.

We have now clarified that diagnosis was primarily clinical and that ancillary investigations were performed based on physician judgment and resource availability.

Manuscript revision made to expanded the “Data Collection” and “Variables” sections to explain that diagnostic evaluation followed routine clinical assessment and was resource-dependent.

**Comment 3:** “What were the process for sending diagnostic tests? Does histopathology and culture of the amputee specimens were done to confirm the diagnosis?”

**Response:** As this was a retrospective registry-based study, the researchers extracted only documented information. The exact procedural workflow for sending specimens (e.g., laboratory referral pathways) was not consistently documented in patient charts. Histopathology and culture were performed in a limited number of cases, but documentation of routine histopathological confirmation of amputated specimens was inconsistent.

We have clarified this methodological limitation in the manuscript.

**Comment 4:** “Mention the duration of medical options as a variable in method?”

**Response:** We acknowledge this important point. However, due to inconsistent documentation of treatment start and stop dates in many charts, duration of medical therapy could not be reliably extracted as a standardized variable. Therefore, it was not included in the regression model.

We have now clarified this in the Methods and acknowledged it as a limitation.

**Comment 5:** “Explain the severity impairment criteria in methods, how it was classified as severe, moderate and mild.”

**Response:** Thank you for highlighting this. The severity classification was based on documented functional status in clinical records:

- Mild impairment: Swelling without documented bone involvement and no limitation in ambulation.
- Moderate impairment: Documented bone involvement and/or limitation in mobility without major deformity.
- Severe impairment: Marked deformity, extensive bone destruction, or documented major limitation in mobility.

We have now clearly described these operational definitions in the Methods section.

**Comment 6:** “Specify in the methods what were the clinical factors for limb loss? e.g., Bone involvement, massive and advance lesion, failed medical treatment, severe superimposed bacterial infections, patient disability, recurrence after surgery.”

**Response:** We agree that specifying predefined clinical factors improves clarity. We have now explicitly listed candidate predictors for amputation in the Methods section, including:

- Bone involvement (X-ray documented)
- Duration of illness
- Prior medical or surgical treatment
- Documented recurrence
- Severe functional impairment

However, factors such as “failed medical treatment” or “superimposed bacterial infection” were not consistently documented and therefore could not be reliably analyzed.

We have clarified which predictors were assessed and which were unavailable due to documentation gaps.

**RESULTS SECTION**

**Comment 1:** No need for Figure 2.

**Response:**
We appreciate this suggestion. Figure 2 has been removed and corresponding information has now been fully incorporated into the Results text in narrative form.

**Comment 2:** What were the pathogens that were positive in microbiology culture?

**Response:** Upon re-examination of the medical records, we found that specific pathogen-level identification was not documented in either the histopathology or microbiology reports. The records only classified cases broadly as “eumycetoma” or “actinomycetoma” without naming specific species. We have clarified this limitation in the Results section and acknowledged it in the Discussion.

**Comment 3:** Elaborate on histopathological findings (e.g., bone involvement and tissue reactions).

**Response:** We appreciate this request for clarification. Bone involvement was documented primarily through X-ray findings (yes/no), rather than histopathological reports. Histopathology records in this retrospective dataset only documented whether a biopsy was performed and whether the diagnosis was consistent with mycetoma; detailed descriptions of tissue reaction, granuloma type, or extent of bone invasion were not available in the files reviewed.

We have clarified this in the Results and Methods sections to avoid overinterpretation of the histopathology findings.

**Comment 4:** Multivariate logistic regression – contradiction regarding occupation.

“Merchant occupation was the only factor significantly associated with delayed care-seeking.”

**Response:** Thank you for identifying this inconsistency. This was indeed an error in the interpretation statement. We confirm that farmer occupation (reference category comparison) was the only factor significantly associated with delayed presentation. The Results section has been corrected accordingly

**Comment 5:** Objective stated early post-operative outcomes and complications, but no results presented.

**Response:** Although documentation of early post-operative outcomes and complications was included as an objective in the original proposal, the retrospective review revealed that such information was not consistently recorded in patient charts. As a result, we were unable to reliably analyze or report these outcomes. I have added the correction on the study methodology limitation.

**CONCLUSION**

**Comment:** Conclusion is appropriate, but discussion should be more policy relevant to support Ethiopian Ministry policy development.

**Response:** We thank the reviewer for this valuable recommendation. We have revised the Discussion and Conclusion sections to strengthen their policy relevance.
